# Evaluation of acute toxicity and gastroprotective activity of *curcuma purpurascens* BI. rhizome against ethanol-induced gastric mucosal injury in rats

**DOI:** 10.1186/1472-6882-14-378

**Published:** 2014-10-06

**Authors:** Elham Rouhollahi, Soheil Zorofchian Moghadamtousi, Omer Abdalla Ahmed Hamdi, Mehran Fadaeinasab, Maryam Hajrezaie, Khalijah Awang, Chung Yeng Looi, Mahmood Ameen Abdulla, Zahurin Mohamed

**Affiliations:** Department of Pharmacology, Pharmacogenomics Laboratory, Faculty of Medicine, University of Malaya, 50603 Kuala Lumpur, Malaysia; Institute of Biological Sciences, Faculty of Science, University of Malaya, 50603 Kuala Lumpur, Malaysia; Department of Chemistry, University of Malaya, 50603 Kuala Lumpur, Malaysia; Department of Biomedical Science, Faculty of Medicine, University of Malaya, 50603 Kuala Lumpur, Malaysia

**Keywords:** *Curcuma purpurascens*, Zingiberaceae, Acute toxicity, Gastric ulcer, Ethanol, Omeprazole

## Abstract

**Background:**

*Curcuma purpurascens* BI. is a medicinal plant from the Zingiberaceae family, which is widely used as a spice and as folk medicine. The aim of the present study is to investigate the gastroprotective activity of *C. purpurascens* rhizome hexane extract (CPRHE) against ethanol- induced gastric ulcers in rats.

**Methods:**

Acute toxicity test was carried out on 36 rats (18 males and 18 females) with low dose of CPRHE (1 g/kg), high dose of CPRHE (2 g/kg) and vehicle (5% Tween 20). To determine the gastroprotective effect of CPRHE, gastric juice acidity, gross and histological gastric lesions, mucus content and ulcer index were evaluated in ethanol-induced ulcer in rats. In addition, superoxide dismutase activity, nitric oxide level and immunohistochemical evaluation of Bax and HSP70 proteins were examined.

**Results:**

The CPRHE acute toxicity test on rats did not reveal any signs of mortality and toxicity up to 2 g/kg. The oral administration of CPRHE at doses of 200 mg/kg and 400 mg/kg and omeprazole (positive control) at a dose of 20 mg/kg to rats remarkably attenuated gastric lesions induced by ethanol. Pre-treatment of rats with CPRHE significantly replenished the depletion of mucus content caused by ethanol administration and decreased the acidity of gastric walls. Further examination of gastric mucosal homogenate revealed significant elevation of superoxide dismutase and nitric oxide activities and reduction in malondialdehyde level in CPRHE-treated group, compared to the lesion control group. Histological assessment of gastric walls obtained from rats pre-treated with CPRHE demonstrated a noteworthy decrease in hemorrhagic mucosal lesions. Immunohistochemical staining showed down-regulation of Bax protein and up-regulation of Hsp70 protein.

**Conclusion:**

Taken together, these findings confirmed the gastroprotective effect of *Curcuma purpurascens* rhizome against gastric damage.

## Background

Ulcer is a lesion or open sore which generally pertains to the mucous membrane or skin of the body. In the digestive system, peptic ulcer in the lining of the duodenum or stomach is an irritating disease which has afflicted a noticeable proportion of the world population [1]. The disruption in the protective effect of the stomach mucosa against gastric acid is a common cause of peptic ulcer [2]. Studies have shown that the etiology of this disorder is related to cigarette smoking, stress, infections, nutritional deficiencies and alcohol consumption [3]. *Helicobacter pylori* infection in particular, contributes to the occurrence of 90% of duodenal ulcers and 80% of gastric ulcers [4]. In addition, the administration of non-steroidal anti-inflammatory drugs remains as one of the drug-related causes of peptic ulcer disease [5]. In recent years, exposure of human to a variety of noxious chemicals and agents has significantly elevated the risk of gastric attacks [6]. Numerous anti-ulcer agents, which are presently being used in the market, exhibit limited efficacy and considerable severe side effects on the human body [7]. Therefore, screening for new agents capable of treating peptic ulcers needs to continue in order to find compounds with reduced side effects while maintaining high-therapeutic efficacy.

Development of pharmaceutical products relies substantially on nutraceuticals, including an extensive range of categories such as spices, herbal products, dietary supplements and functional foods [8]. Numerous plants from a variety of taxonomic families have been studied for their anti-ulcer activities [5, 9–11]. One such taxonomic family with extensive medicinal uses against gastric mucosal lesions is Zingiberaceae. Numerous plant species in this family, such as *Boesenbergia rotunda* [9], *Curcuma longa* [12], *Zingiber officinale* [7], *Alpinia galanga* [13] and *Elettaria cardamomum* [14] have been elucidated for their gastroprotective properties.

*Curcuma purpurascens* BI. is a member of the Zingiberaceae family. This plant originally grew in Indonesia and its rhizome is used as a spice [15]. The *C. purpurascens* plant commonly known also as “Temu tis” and “Koneng tinggang” has been reported to have extensive traditional uses in rural communities, similar to other *curcuma* species [15, 16]. Rhizome of this plant has been used in folk medicine for the treatment of wounds, scabies, itch, fever, cough and boils. The mixture of the rhizomes with *Alyxia stellata* is used as a poultice after childbirth [15]. Despite these traditional applications, there is no scientific investigation for the potential bioactivities of this plant. Since natural products with a long history of folk application can provide new therapeutic approaches for the treatment of various diseases and ailments [17], the current study is conducted to evaluate the acute toxicity and gastroprotective activity of the *C. purpurascens* rhizome against ethanol-induced ulceration in rats.

## Methods

### Chemical and drugs

In this study, omeprazole as the reference anti-ulcer medicine was obtained from the University of Malaya Medical Center and dissolved in 10% Tween 20 (Merck, Germany). A dilution of Tween 20 (5% v/v) was used as the vehicle.

### Sample collection

The *C. purpurascens* rhizome was collected from Yogyakarta, Indonesia. The botanical identification was made by Mr. Teo Leong Eng., Faculty of Science, University of Malaya. A voucher specimen KL 5793 was deposited in the herbarium of the Department of Chemistry, Faculty of Science, University of Malaya, Kuala Lumpur, Malaysia.

### Preparation of the extract

The air-dried and powdered rhizomes (1.0 kg) were macerated with n-hexane at room temperature for three days. The resulting filtrate of the rhizome was concentrated by a rotary evaporator at 40°C and stored at -20°C until use. The *C. purpurascens* rhizome hexane extract (CPRHE) was dissolved in carboxymethylcellulose for *in vivo* animal study.

### Gas chromatography of CPRHE

The analysis of the hexane extract was performed using an Agilent and LECO RESTEK, Rxi-5MS capillary column (30 m, 0.25 mm i.d., 0.25 μm film thickness) and a mass spectrometer Pengasus HT High Throughput TOFMS, as previously described [18]. Compounds were identified from their mass spectra, by comparison of the retention times of peaks with interpretation of MS fragmentation patterns from data library.

### Animals and ethical issues

Healthy female and male *Sprague Dawley* rats (150–180 g, 6–8 weeks old) were obtained from the Animal Experimental Unit, Faculty of Medicine, University of Malaya, Kuala Lumpur, Malaysia. The animals were housed in an isolated cabin under controlled conditions of temperature (~24°C), humidity (~50%) and light (a daily ratio of 1:1). The animals were allowed access to standard rat pellets and RO water. All the animal experiments were carried out in compliance with the National Institutes of Health Guide for the care and use of Laboratory Animals and with the prior approval from the committee of Animal House, Faculty of Medicine, University of Malaya (Ethics No. 2014-03-05/PHAR/R/ER).

### Acute toxicity study

To determine a safe range of doses for CPRHE, toxicity evaluation of CPRHE was carried out as previously described [19]. In brief, 36 rats, including 18 males and 18 females were divided into three groups labeled as the vehicle (5% Tween 20), low dose of CPRHE (1 g/kg) and high dose of CPRHE (2 g/kg). The rats were fasted for 16 h prior to the dosing (water was accessible except for the last 2 h). Following the dosing, food was withheld for another 1 to 3 h. Any other signs of toxicities and mortality were further recorded during the period of two weeks. On day 15, the animals were sacrificed for hematological and histological analysis.

### Ethanol-induced gastric ulceration

The preventive potential of CPRHE against superficial hemorrhagic mucosal lesions were investigated in the normal rats. Prior to the experiment, *Sprague Dawley* male rats were fasted for 24 h (water was accessible except for the last 2 h). Thirty rats were divided randomly into 5 groups of 6 rats each and pre-treated accordingly (Table 1). After 1 h of pre-treatment, all the rats were gavaged with 5% Tween 20 (5 ml/kg) or absolute ethanol (5 ml/kg) based on the animal experimental design. The rats were sacrificed 1 h later with an over-dose of xylazine and ketamine and their stomachs were immediately excised.

**Table 1:** **The experimental design and specifications of the animal study**

Groups	Description	Pre-treatment	Treatment
Group A	Normal control	5% Tween 20 (5 ml/kg)	5% Tween 20
Group B	Lesion control	5% Tween 20 (5 ml/kg)	absolute ethanol
Group C	Treatment control	Omeprazole (20 mg/kg)	absolute ethanol
Group D	Experimental group1	CPRHE (200 mg/kg)	absolute ethanol
Group E	Experimental group2	CPRHE (400 mg/kg)	absolute ethanol

### Determination of the loss in mucosal content and gastric juice acidity

The stomach of each rat was dissected along the greater curvature, and pH-meter titration with 0.1 N NaOH was used to analyze the hydrogen ion concentration in the gastric contents expressed in mEq/I value. Then, a glass slide was applied to gently scrape the gastric mucosa of the rats followed by the weighing of the obtained mucus with a precision electronic balance.

### Macroscopic analysis of lesions

In accordance with several studies, ethanol-induced ulcers on the gastric mucosa were characterized as elongated bands of hemorrhagic lesions parallel to the long axis of the stomach [11, 20]. The hemorrhagic damage of the stomach was determined by assessment of luminal surface. The protective potential (P%) of each pre-treatment was calculated using a planimeter (10 × 10 mm^2^) and dissecting microscope (1.8×) where UC and UT were the ulcer area of the control and treated group, respectively. The measurement of ulcer area was performed as previously described in detail by Abdelwahab et al. [9].


### Histological evaluation of gastric lesions

Histological analysis of the specimens of the gastric walls was carried out using 10% buffered formalin for the fixation. The samples were embedded in paraffin followed by the 5 μm sectioning and staining with hematoxylin and eosin.

### Determination of lipid peroxidation activity using the thiobarbituric acid reactive substance assay

To measure malondialdehyde (MDA) concentration, we carried out thiobarbituric acid reactive substance (TBARS) assay as described previously [21]. In brief, the homogenated stomach (10% w/v) in 0.1 mol/l PBS was centrifuged at 4°C for 10 min. Then, the supernatant (3 ml) was mixed with 20% trichloroacetic acid solution and 0.67% 2-thiobarbituric acid followed by heating in a water-bath (95°C) for 30 min. Next, the MDA concentration of the obtained supernatant was determined spectrophotometrically at 532 nm. The protein concentrations were expressed as MDA μmol/g protein using the Lowry method [22].

### Determination of superoxide dismutase (SOD) activity

The activity of SOD enzyme was estimated by determining its potential to suppress the photochemical reaction of NBT (nitroblue tetrazolium), as previously described by Sun and colleagues [23]. In this assay, the homogenated tissues were centrifuged twice at 4°C for 10 and 20 min. In a dark chamber, the reactant (1 ml, 50 mM phosphate buffer, 100 nM EDTA and 13 mM l-methionine, pH 7.8) was mixed with the resulting supernatant (30 μl), NBT (150 μl, 75 μM) and riboflavin (300 μl, 2 μM). The resulting solution was exposed to fluorescent light bulbs (15 W) for 15 min and the absorbance was determined at 560 nm wavelength using a spectrophotometer.

### Nitric oxide level

To evaluate the effect of CPRHE on nitric oxide (NO) generation in the gastrointestinal tract, Griess reaction was used to determine total nitrite/nitrate levels, as previously described by Tsikas and colleagues [24]. In brief, the supernatant (50 μl) of the homogenated stomach was mixed with the Griess reagent. After 10 min, the subsequent colorimetric analysis was carried out at 540 nm using a Tecan Infinite 200 Pro microplate reader (Tecan, Männedorf, Switzerland). The generated NO in the culture supernatant was determined using a standard curve of sodium nitrite and results were expressed as μmoles nitrite/nitrate per gram of protein.

### Immunohistochemical evaluation

Immunohistochemical analysis of Bax and HSP70 proteins was carried out as previously described with some modifications [25]. In brief, tissues were paraffinized in xylene and rehydrated using graded alcohol. The boiling sodium citrate buffer (10 mM) was used for antigen retrieval process followed by immunohistochemical staining according to the manufacture’s instruction (Dakocytomation, USA). After blocking the endogenous peroxidase with peroxidase block, Bax (1:200) or Hsp70 (1:500) biotinylated primary antibodies were applied and the sections were incubated for 15 min. Next, sections were incubated with the appropriate amount of streptavidin–HRP for 15 min. Next, the sections were exposed to diaminobenzidine substratechromagen for 5 s, then dipped in weak ammonia (0.037 mol/L) for ten times. Brown stains on the slides indicated positive findings of the immunohistochemical staining as observed under a light microscope.

### Statistical analysis

All values were reported as mean ± SD. The statistical significant differences between groups were determined using one-way ANOVA followed by post hoc Tukey’s multiple comparison test. A value of *p* <0.05 was considered significant.

## Results

### Gas chromatography profile of CPRHE

**Figure 1:**
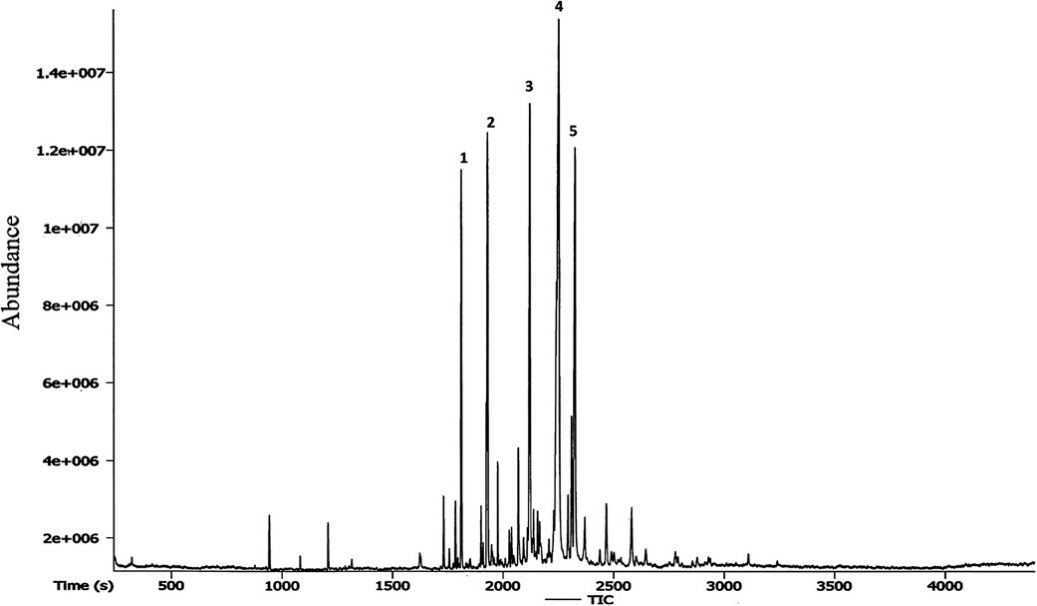
**A typical gas chromatogram.** The chromatogram showed the chemical constituents of *C. purpurascens* hexane extract.

As shown in Figure 1, the hexane extract was characterized by use of GC-MS-TOF. The chromatogram showed that the major compounds in CPRHE are c-elemene (1), benzofuran, 6-ethenyl-4,5,6,7-tetrahydro-3,6-dimethyl-5-isopropenyl (2), 3,7-cyclodecadien-1-one,3,7-dimethyl-10-(1-methylethylidene) (3), turmerone (4) and curlone (5) (Table 2).

**Table 2:** **The major bioactive compounds of** ***C. purpurascens*** **hexane extract as characterized using GC-MS-TOF analysis**

Peak No.	Name of Compounds	Retention Time (s)	Mass
1	c-elemene	1812.55	189
2	benzofuran, 6-ethenyl-4,5,6,7-tetrahydro-3,6-dimethyl-5-isopropenyl	1931.3	216
3	3,7-cyclodecadien-1-one,3,7-dimethyl-10-(1-methylethylidene)	2123.3	218
4	turmerone	2254.45	218
5	curlone	2326.55	218

### Acute toxicity study

**Figure 2:**
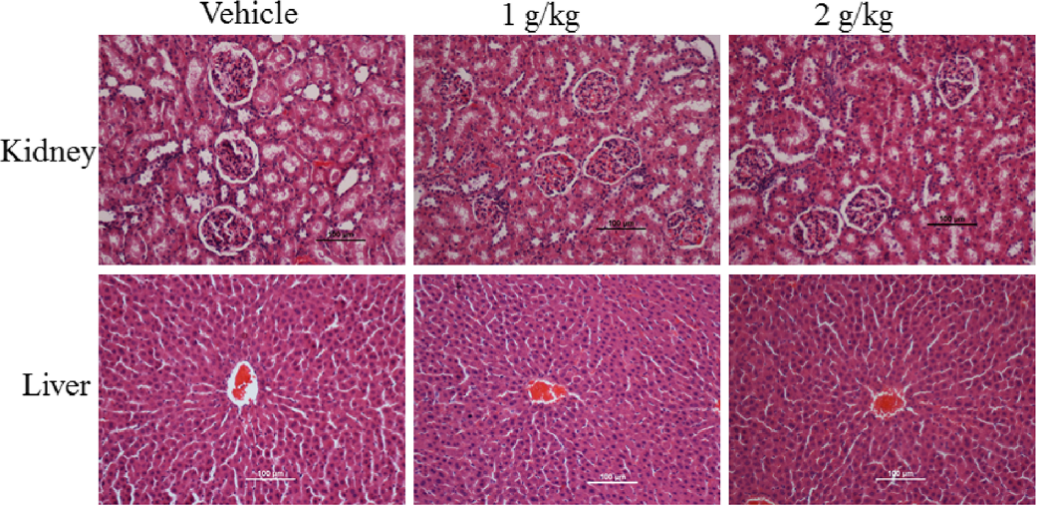
**Histological sections.** Histopathology of kidney (first row) and liver (second row) in acute toxicity study representing the rats treated with vehicle (5% Tween 20), CPRHE (1 g/kg) and CPRHE (2 g/kg). The result did not show significant differences in the structures of kidney and liver between treated and control groups (20× magnifications).

In the acute toxicity study, all rats survived and did not manifest any sign of toxicity and abnormality at 1 and 2 g/kg dosage. For a duration of 14 days, there was no behavioral or body weight changes and no abnormal signs were observed. As shown in Table 3, the serum biochemical parameters were reported to be normal. Hematological analysis of kidney and liver did not elicit any noteworthy changes in the treated group compared to the control group (Figure 2). The 50% oral lethal dose (LD_50_) for the male and female rats was greater than 2 g/kg body weight.

**Table 3:** **Serum biochemical analysis of rats. Effect of CPRHE on (a) renal function test, (b) liver function test and (c) hematology analysis of the rodents**

Animal groups	Serum biochemical analysis								
	**(a) Kidney biochemical parameters**								
	**Sodium (mM/L)**	**Potassium (mM/L)**		**Chloride (mM/L)**		**Co**_**2**_ **(mM/L)**	**Anion (mM/L)**	**Urea (mM/L)**	**Creatinine (** ***μ*** **M/L)**
Vehicle	142.23 ± 0.45	4.85 ± 0.06		104.59 ± 0.45		23.89 ± 0.64	19.36 ± 0.48	5.28 ± 0.58	30.75 ± 1.87
CPRHE (1 g/kg)	143.54 ± 0.37	5.12 ± 0.72		106.24 ± 0.54		21.49 ± 0.73	19.58 ± 0.75	5.93 ± 0.43	29.81 ± 2.27
CPRHE (2 g/kg)	143.85 ± 0.82	4.91 ± 0.034		105.27 ± 0.56		22.85 ± 0.48	19.95 ± 0.39	5.68 ± 0.37	31.46 ± 2.19
**Animal groups**	**(b) Liver biochemical parameters**								
	**Total protein (g/L)**	**Albumin (g/L)**	**Globulin (g/L)**	**TB (** ***μ*** **mol/L)**	**CB (** ***μ*** **mol/L)**	**AP (IU/L)**	**ALT (IU/L)**	**AST (IU/L)**	**GGT (IU/L)**
Vehicle	60.76 ± 0.94	9.56 ± 0.39	51.07 ± 1.28	2.19 ± 0.13	0.87 ± 0.17	154.45 ± 5.37	50.49 ± 1.34	173.82 ± 5.38	3.45 ± 0.27
CPRHE (1 g/kg)	58.37 ± 0.57	8.35 ± 0.53	50.68 ± 1.32	2.14 ± 0.16	0.91 ± 0.14	153.23 ± 5.87	45.65 ± 1.74	175.48 ± 6.49	3.37 ± 0.53
CPRHE (2 g/kg)	59.67 ± 1.29	8.87 ± 0.19	50.25 ± 1.45	2.15 ± 0.14	0.84 ± 0.16	155.73 ± 6.82	46.76 ± 1.48	176.27 ± 6.52	3.57 ± 0.43
**Animal groups**	**(c) Hematological parameters**								
	**HGB (g/dL)**	**HCT (%)**	**RBC (10** ^**6**^ **/μL)**	**MCV (fL)**	**MCH (pg)**	**MCHC (g/dL)**	**RDW (%)**	**WBC (10** ^**3**^ **/μL)**	**Platelet (10** ^**3**^ **/μL)**
Vehicle	15.03 ± 0.13	46 ± 0.00	9.38 ± 0.13	59.12 ± 0.65	19.25 ± 0.31	34.22 ± 0.19	17.03 ± 0.44	6.12 ± 0.47	986.75 ± 23.65
CPRHE (1 g/kg)	15.47 ± 0.13	46 ± 0.00	9.64 ± 0.18	58.54 ± 0.63	18.89 ± 0.36	34.04 ± 0.33	18.23 ± 0.58	6.23 ± 0.34	995.36 ± 25.32
CPRHE (2 g/kg)	15.98 ± 0.12	46 ± 0.00	9.83 ± 0.16	57.78 ± 0.76	18.59 ± 0.29	33.93 ± 0.28	18.77 ± 0.34	6.31 ± 0.39	1011.52 ± 23.18

Values expressed as mean ± SEM. ALT, alanine aminotransferase; AST, aspartate aminotransferase; AP, alkaline phosphatase; CB, conjugated bilirubin; GGT: G-glutamyl transferase; HCT, hematocrit; HGB, hemoglobin; MCH, mean corpuscular hemoglobin; MCHC, mean corpuscular hemoglobin concentration; MCV, mean corpuscular volume; RBC, red cell count; RDW, red cell distribution width; TB: total bilirubin; WBC, white cell.

The results did not show any significant difference between groups.

### pH of gastric content and determination of mucus production

The acidity of gastric content in rodents administered orally with ethanol was significantly increased compared to the normal control group, as shown in Table. 4. After treatment with omeprazole (positive control), the acidity was significantly attenuated (*P <*0*.*05) and animals pre-treated with CPRHE at high dose and low dose elicited significant (*P <*0*.*05) elevation of pH. The gastric mucus content was significantly depleted in animals pre-treated with ethanol. Meanwhile, omeprazole and CPRHE treatment significantly replenished the loss in mucosal content *P <*0*.*05, Table 4). The findings of both parameters suggested the anti-ulcer effect of CPRHE.

**Table 4:** **Gastroprotective effect of CPRHE against ethanol-induced gastric injury**

Animal Groups	Animal Groups	pH of Gastric tissue	Mucus Weight (g)	Ulcer area (mm) ^2^	Inhibition (%)	MDA (μmol/g protein)	SOD (U/g protein)	Nitric oxide (μmol/g protein)
A	Normal control	7.02 ± .004	0.89 ± 0.03	**-**	**-**	10 ± 1.1	528 ± 16.51	10 ± 1.3
B	Lesion control	2.99 ± 0.17	0.41 ± 0.01	880 ± 16.23	**-**	28 ± 2.98	333 ± 9.98	5.9 ± 0.47
C	Treatment control	6.14 ± 0.24*	0.79 ± 0.03*	195 ± 8.97*	77	13 ± 0.71*	481 ± 12.22*	9.1 ± 1.4*
D	CPRHE (200 mg/kg)	4.47 ± 0.09*	0.68 ± 0.02*	455 ± 12.24*	48	21 ± 0.22*	393 ± 7.67*	7.6 ± 0.8*
E	CPRHE (400 mg/kg)	5.82 ± 0.41*	0.72 ± 0.02*	325 ± 10.67*	63	17 ± 0.41*	459 ± 10.73*	8.4 ± 0.9*

All values are expressed as mean ± SD. *indicates (*P* <0.05) compared to the (B) lesion control group. CPRHE: *C. purpurascens* rhizome hexane extract.

### Macroscopic evaluation of gastric lesions

**Figure 3:**
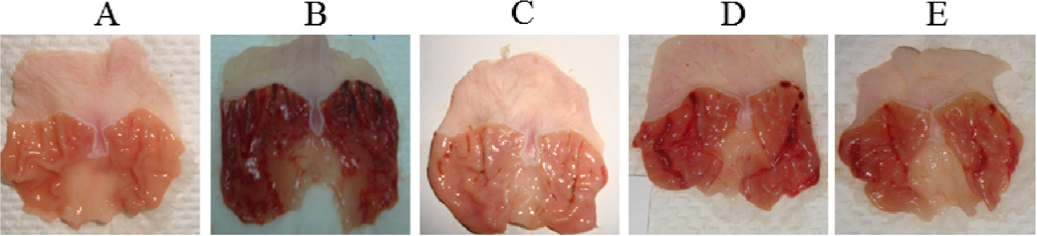
**Gross evaluation.** Results demonstrated that the rodents pre-treated with **(C)** omeprazole and CPRHE at doses of **(D)** 200 mg/kg and **(E)** 400 mg/kg had conspicuously decreased area of gastric ulcer formation compared to **(B)** ulcer control. **(A)** Normal control group demonstrated no gastric lesion formation.

The administration of ethanol to the rats induced noticeable black hemorrhagic lesions in the gastric walls (Figure 3B) with ulcer area of 880 ± 16.23 (mm)^2^ (Table 4). As shown in Figure 3, rodents pre-treated with CPRHE (Figure 3D and E) and omeprazole (Figure 3C) had markedly reduced areas of gastric ulcer formation in comparison with lesion control group (Figure 3B). Pre-treatment of rats with CPRHE at doses of 200 mg/kg and 400 mg/kg suppressed the ulcer area formation to 455 ± 12.24 (mm)^2^ and 325 ± 10.67 (mm)^2^, which was comparable to the suppressive effect of omeprazole 195 ± 8.97 (mm)^2^. The incidence of ulcer was decreased by 48% and 63% after treatment with CPRHE at doses of 200 mg/kg and 400 mg/kg, respectively (Table 4). It is worthy to note that CPRHE treatment helped to flatten the gastric mucosal folds in rodents (Figure 3D and E).

### Histological assessment of gastric lesions

**Figure 4:**
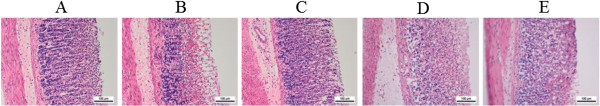
**Histological evaluation.** Histopathology of rodents pre-treated with **(C)** omeprazole or CPRHE at the doses of **(D)** 200 mg/kg and **(E)** 400 mg/kg compared to the **(B)** lesion control group. **(A)** Normal control group demonstrated normal histological structure. (H and E stain10×).

Microscopic analysis of the gastric lesions in the ulcer control group elicited extensive damage to the gastric mucosa of the rodents characterized by disrupted surface epithelium and deeply penetrated necrotic lesions into mucosa associated with conspicuous leucocytes infiltration and severe edema of submucosal layer (Figure 4B). Pretreatment with CPRHE (Figure 4D and E) and omeprazole (Figure 4C) revealed a protective effect by reduction in leucocytes infiltration and submucosal edema. The replenishment of the loss in mucus content by CPRHE was evidenced by the stumpy amount of magenta color in the histological analysis as compared to the control group (Figure 4D and E). The results showed that CPRHE pre-treatment at high dose and low dose markedly disrupted ethanol-induced destruction of gastric mucosa.

### Assessment of stomach malondialdehyde and superoxide dismutase

To determine the effects on lipid peroxidation and oxidative stress, we determined the level of MDA in gastric tissue homogenate. After treatment with ethanol, the MDA level (28 ± 2.98 μmol/g) was significantly (*P <*0*.*05) elevated compared to the normal control group (10 ± 1.1 μmol/g). The results showed that administration of CPRHE and omeprazole before ethanol significantly (*P <*0*.*05) decreased the MDA level compared to the lesion control group (Table 4). Ethanol treated rodents elicited a significantly lower SOD activity compared to the normal control group (*P <*0*.*05), which was elevated upon treatment with CPRHE (Table 4).

### Assessment of nitric oxide level

The perturbation in nitric oxide levels of the stomach was investigated using Griess reagent, as shown in Table 4. In gastric tissue homogenate, the nitric oxide level in lesion control group was markedly (*P <*0*.*05) lower (5.9 ± 0.47 μmol/g protein) compared to the normal control group (10 ± 1.3 μmol/g protein). Administration of CPRHE significantly (*P <*0*.*05) elevated nitric oxide level, which was comparable to the nitric oxide-generation effect of omeprazole.

### Immunohistochemistry

**Figure 5:**
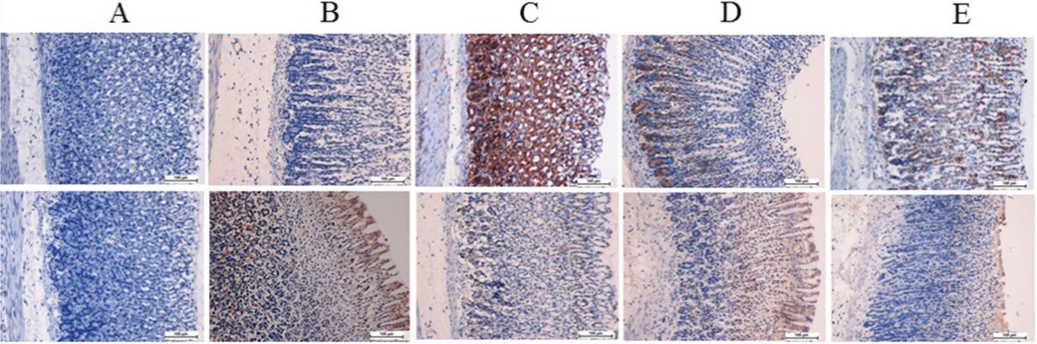
**Immunohistochemical examination of Hsp70 (first row) and Bax (second row).** The results depicted the down-regulation of Bax and up-regulation of Hsp70 proteins in rats pre-treated with **(C)** omeprazole and CPRHE at the doses of **(D)** 200 mg/kg and **(E)** 400 mg/kg compared to the **(B)** lesion control group. **(A)** Normal control group demonstrated normal Immunohistochemical structure.

Immunohistochemical analysis of the gastric injuries revealed that pre-treatment with CPRHE at the doses of 200 mg/kg and 400 mg/kg caused conspicuous up-regulation of Hsp70 protein when compared with the ulcer control group (Figure 5). In addition, the expression of Hsp70 in normal control group was relatively higher than the expression in ulcer control group. Immunohistochemical examination of Bax protein indicated a down-regulation of this protein after administration of CPRHE and omeprazole. Meanwhile, the expression of Bax was up-regulated in ulcer control group in comparison with normal control group.

## Discussion

Numerous species of Zingiberaceae family, such as *Curcuma phaeocaulis, Curcuma longa*, *Amomum villosum, Zingiber officinale, Alpinia oxyphylla* and *Alpinia officinarum* are used as folk medicine by the natives from various countries. Traditional uses and scientific inspections of these plants have confirmed the safety of these plants as complementary medicine [26–29]. In the present study, the acute toxicity of *C. purpurascens* was evaluated in rats. Our findings showed no sign of renal or hepatic toxicity as investigated by biochemical and histological analysis.

One of the contributing factors in gastric ulcer formation is ethanol. Due to the rapid penetration of ethanol into the gastric mucosa, it is widely utilized to induce experimental gastric ulcer in numerous *in vivo* studies [29–31]. The elevation in mucosal permeability and release of vasoactive products by ethanol result in vascular damage and gastric cell necrosis prior to the ulcer formation. Furthermore, it is believed that the generation of reactive oxygen species (ROS) by ethanol has a significant role in ulcer formation [32]. Therefore, in our study, the administration of ethanol to rats was used to induce gastric lesions.

Our findings in this study showed that CPRHE can effectively suppress gastric acidity and also suppress the destruction of gastric wall mucus. It was reported earlier that herbal products could elevate the gastro-defensive system, especially gastric wall mucus secretion in patients with gastric ulcer [33]. The depletion of mucus secretion is one of the pathogenic mechanisms accountable for gastric mucosal erosions [34]. Previous studies have explained the close correlation between suppression of gastric acidity and effectiveness of treatment. The ability to attenuate the gastric acid secretion is considered to be the mainstay of treatment for gastric ulceration [35, 36]. One of the standard drug, omeprazole, has shown remarkable healing rates among patients with peptic ulcer because of its ability to minimize the degree of gastric acidity through inhibition of the proton pump [37].

The perturbation in balance between gastro-protective mechanisms and gastrotoxicity of different agents is the basis of acute inflammation and secretion of several proinflammatory cytokines [38]. It is reported earlier that acute inflammation induced by ethanol is accompanied by neutrophils infiltration of gastric wall mucus [39]. As shown in Figure 4, our results demonstrated that submucosal infiltration was effectively suppressed by pre-treatment of rats with CPRHE. An extensive generation of ROS and free radicals causes metabolic impairments and irreversible cell damages in the human body [40]. As such, protecting the gastric tissue from oxidative damages can provide successful treatment approaches by natural products against ulcer formation [41]. Superoxide dismutase by converting the superoxide to hydrogen peroxide has a critical role in this protecting effect [42]. Superoxide radical anions such as ROS are generated by neutrophils, which results in the reaction with cellular lipids and the production of lipid peroxides [43]. An effective indicator of oxidative stress and mucosal injuries by ROS is malondialdehyde (MDA), which is a major metabolite of lipid peroxidation [44]. This is the first study to show that oral administration of CPRHE could protect against gastric ulceration by elevating superoxide dismutase activity, which is reflected by decreased MDA production.

In our study, the NO level is significantly increased after pre-treatment with CPRHE. The important role of NO synthesis for the gastroprotective system and effectiveness of anti-ulcer agents has been elucidated earlier [45]. It has been shown that suppression of NO pathway by L-NAME markedly inhibited the gastroprotective activity of several anti-ulcer medications. Furthermore, formation of ethanol-induced gastric lesions is remarkably abolished by NO-stimulating drugs. Meanwhile, reduction in NO synthesis can increase the susceptibility of the gastric mucosa to the destructive effects of ethanol [46, 47]. Therefore, increasing NO level by CPRHE treatment is beneficial in alleviating ethanol-induced gastric wall destruction as shown in this study.

Our findings showed that administration of CPRHE to rats induced the up-regulation of Hsp70 protein associated with down-regulation of Bax protein. Hsp70 is a 70 kDa protein which belongs to the heat shock protein family. This protein is abundantly produced in response to various forms of stress, such as toxic agents, oxidative stress, infection and heat shock [48]. It is well known that the ethanol-triggered generation of ROS could suppress Hsp70 expression and intensifies the oxidative damages [49]. Hence, natural products with the ability to induce the over-expression of Hsp70 may provide higher threshold of protection against gastric lesions [50]. Previous studies have demonstrated that the suppression of Bax expression or its dysfunction can protect cells against programmed cell death through prevention of cytochrome *c* release from mitochondria to cytosol. A variety of stimuli, including heat, radiation and excessive production of ROS cause the dimerization of Bax protein and translocation to the outer mitochondrial membrane, which trigger cytochrome *c* release [51–53]. In our study, suppression of ROS production induced by CPRHE seems to be the reason for the attenuation of Bax expression.

Previous study done using *Curcuma longa* showed that hexane soluble fraction of the rhizome was effective against HCl-induced peptic ulcer, while the hexane non-soluble fraction was inactive [54]. Therefore, in this study, the hexane extract was used to investigate the anti-ulcer potential of *C. purpurascens* rhizome. Furthermore, gas chromatography profile of CPRHE suggested the presence of turmerone as the major active compound. Turmerone is an active constituent with powerful antioxidant activity, which has been previously isolated from other *Curcuma* species [55]. A previous clinical trial suggested that turmerone is responsible for the anti-ulcer effect of *C. longa* [12]. However, insufficient research studies on the photochemistry of this spice could not provide enough evidence to confirm the major role of turmerone for the observed anti-ulcer effect.

## Conclusions

The present study elucidated the anti-ulcer effect of *C. purpurascens* rhizome against ethanol-induced gastric lesions. The gastroprotective mechanism of CPRHE was achieved by increasing SOD and NO levels, which in turn suppressed gastric acidity and prevented the destruction of the gastric mucus wall. Immunohistochemistry analysis of gastric homogenate elicited the critical role of Bax down-regulation and Hsp70 up-regulation. In addition, the acute toxicity test of CPRHE provided useful information regarding the safety of this traditional medicinal plant. Further investigation on the active phytochemical constituents contributing to the gastroprotective activity is undergoing in our laboratory to identify potential anti-ulcer agent in CPRHE.
